# A Novel Frameshift Mutation in Abnormal Spindle-Like Microcephaly (ASPM) Gene in an Iranian Patient with Primary Microcephaly: A Case Report

**Published:** 2019-11

**Authors:** Afsaneh BAZGIR, Mehdi AGHA GHOLIZADEH, Faezeh SARVAR, Zahra PAKZAD

**Affiliations:** Department of Medical Genetics, Fardis Central Lab, Alborz, Iran

**Keywords:** Autosomal recessive primary microcephaly, ASPM, Whole exome sequencing

## Abstract

Autosomal recessive primary microcephaly (MCPH) is a rare genetic disorder, leading to the defect of neurogenic brain development. Individuals with MCPH reveal reduced head circumference and intellectual disability. Several MCPH loci have been identified from several populations. Genetic heterogeneity of this disorder represents molecular testing challenge. An 8 yr old female, born from consanguineous parents, was attended to Fardis Central Lab, Alborz, Iran. Based on the reduced circumference and intellectual disability, MCPH was diagnosed. Whole exome sequencing of the patient identified a novel homozygous frameshift mutation (c.2738dupT, p.Cys914fs) in exon 9 Abnormal Spindle-like Microcephaly (ASPM) gene. By Sanger sequencing, segregation analysis showed that both parents were heterozygous carriers for this variant. The novel frameshift mutation likely truncates the protein, resulting in loss of normal function ASPM in homozygous mutation carriers. The study might add a new pathogenic variant in mutations of the ASPM gene as a causative variant in patients with MCPH and might be helpful in genetic counseling of consanguineous families.

## Introduction

Autosomal recessive primary microcephaly (MCPH) is a genetically heterogeneous condition that is distinguished by reduced head circumference and mental retardation at birth (1). Depending on different population and consanguineous populations, the birth incidence of primary microcephaly varies from 1.3 to 150/100000 (2). To date, 18 genes (MCPH1-MCPH18) have been known to be mutated in MCPH patients (3). The most common gene known in primary microcephaly are mutations in the abnormal spindle-like microcephaly (ASPM) gene at the MCPH5 locus. These mutations include deletions, duplications, single base-pair changes and intronic variants that resulting in frameshift and protein-truncating. (4). ASPM gene has the important role in centriole biogenesis, normal function of mitotic spindle and neocortical development (5). The diagnosis of MCPH is challenging due to the genetic heterogeneity (3). Recently, the utilization of whole exome sequencing (WES) has improved to investigate the genetic etiology of heterogeneous disorders (6).

In this study, we report an 8 years old female with MCPH with a novel homozygous frameshift mutation in exon 9 of the ASPM gene using WES. This variant has not been previously reported in another Iranian family and might be a new possible candidate to describe the patient’s phenotype (7, 8).

## Case presentation

An 8-yr-old female presented to our laboratory (Fardis Central Laboratory, Alborz) at March 2018 with microcephaly, intellectual disability, developmental delay, craniofacial abnormalities, epilepsy, muscle weakness in left arm, attention-deficit/hyperactivity disorder (ADHD) and polyneuropathy. While motor milestones were normal, her cognitive and language skills were delayed. Developmental delay milestone was at 12 to 15 months old by delaying in reaching language and thinking skills. Craniofacial include microcephaly due to craniosynostosis.

**Fig. 1:**
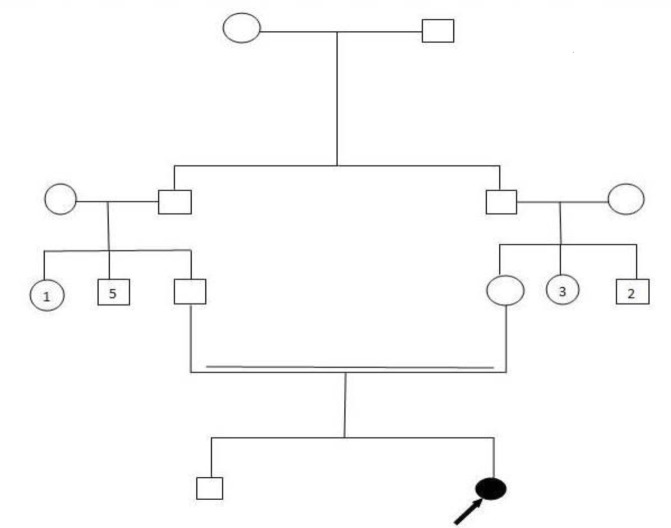
Pedigree and sequencing chromatograph of the studied family. Filled circle represents an affected individual. Double lines show consanguineous

On physical exam at 8 years of age, the patient’s weight was 18 kg, height 113 cm and head circumference 40 cm which was 9 SD below the population age – and sex-related mean. In the case of patients’ family history, the parents were first cousins originating from the Northwest of Iran with normal head circumference and have normal intelligence. There was no history of any genetic disorders including neurological disease in the family (Fig. 1).

Consent form was obtained from the parents earlier to beginning of the examination.

Genomic DNA was extracted from peripheral blood from the proposita and her parents using DNA extraction kit (Roche, USA). Human whole exome enrichment was performed in proposita using Agilent SureSelect V6 Target Enrichment Kit and the library was sequenced on Illumina Hiseq 4000 platform with an average depth of 110.7X for target regions (Macrogen, South Korea).The resulting VCF (Variant Call Format) file contains approximately 107500 variants. The Variants were called using with GATK and filtered to focus analysis on those variants within the microcephaly genes. All exon and flanking 10bp were detected and analyzed.

**Fig. 2:**
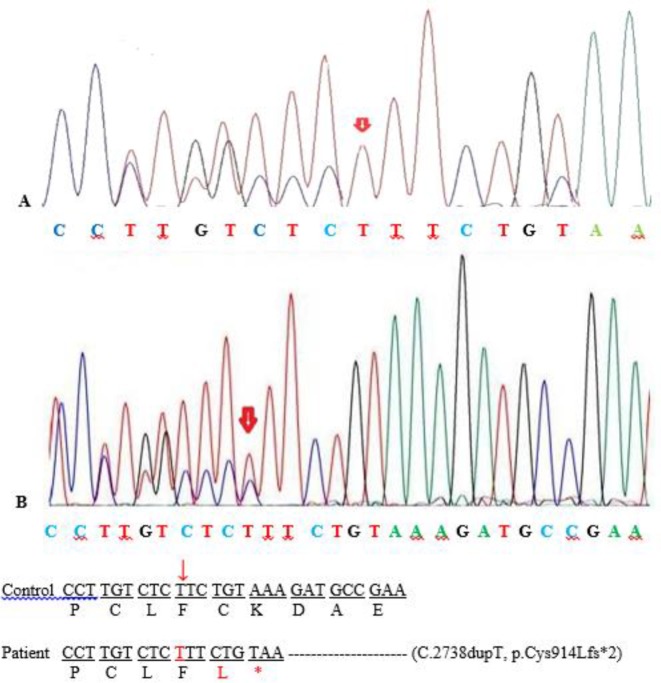
Partial DNA sequence of ASPM gene. The sequence chromatograms showed the homozygous mutation c.2738dupT in the patient (A) and the heterozygous mutation in the parents (B)

Upon of analysis of the exom data, we identified a novel homozygous mutation c.2738dupT; p.Cys914fs in exon 9 of ASPM gene which was confirmed by targeted Sanger sequencing. By Sanger sequencing, segregation analysis showed that both parents are heterozygous carriers for this variant. (Fig. 2). This variant has not been previously reported and it was absent in ExAC and 1000G databases. Based on the ACMG guideline, this variant can be classified as a likely pathogenic variant. In silico computational analysis (Mutation Taster, and Combined Annotation Dependent Depletion (CADD)) predicted that the novel frameshift mutation (p.Cys914Lysfs^*^2) in ASPM can cause premature stop codon, resulting to truncate the protein synthesis.

For confirmation of the mutation detection, Sanger DNA sequencing was performed. Primers were designed through primer3 software for amplification of exon 9 of ASPM gene. Primers used were as follows: 5′TGTGCTTGCTACCCTACACT 3′ (Forward) and 5′ACGAGGGATGGTTAGAATTACTG3' (Reverse). Direct sequencing was carried out using 3130 automated DNA sequencer (Applied Biosystems) and the sequences were analyzed by sequencing analysis software, version 5.3.1.

## Discussion

WES analysis of an Iranian patient with MCPH revealed novel homozygous frame-shift mutation (c.2738dupT) in exon 9 of the ASPM gene. MCPH is a disorder of brain development in which individuals were born with reduced head circumference and mental retardation (1). Eighteen genes have been identified in MCPH, to date. Mutation in the ASPM gene at MCPH5 is the most common cause of MCPH in different population (4). Prevalence of MCPH5 locus differs in different populations. It is reported 13.3% in an Iranian population (7). The ASPM gene located on chromosome 1q31.3 at MCPH5 locus and contains 28 exons and 3,477 amino acids (NM_018136). The ASPM protein has an important role for the normal functioning of the mitotic spindle in embryonic neuroblasts (9). Many of mutations consist of nonsense, frame-shift, deletion, single base pair change, a breakpoint translocation and splice affecting mutations identified in throughout the ASMP gene and most of them result in truncating of protein (9–11). ASPM mutations among an Iranian family with MCPH most likely leading to functional impairment of the gene product (7). Mutations of ASPM can influence of orientation of the mitotic spindle, resulting in the decrease of the brain size and affect the asymmetrical to symmetrical cell division ratios, leading to decrease the neuronal cells (12).

In this study, we identified a novel homozygous frame-shift mutation (c.2738dupT) in exon 9 of the ASPM gene in an Iranian patient with MCPH through WES. This variant has not been previously reported and in silico analysis indicated that this variant could lead to a premature stop codon in 2 codons downstream, resulting in early protein truncation (p.Cys914Lysfs^*^2). Most of the identified mutations in ASPM gene are predicted to produce a truncated protein. No correlation has been observed between the position of the mutation in the gene and degree mental retardation or clinical outcome but in a study showed that patients with p.R1327* mutation had worst growth pattern, IQ and occurrence seizure (13). This may indicate that nonsense-medicated decay is the common mechanism of ASPM mutations. Consistent with previous reports of ASPM mutations, the detected variant is also predicted to induce nonsense-mediated mRNA decay (NMD) and result in loss of normal function of ASPM in homozygous mutation carriers. A few mutations in the downstream of this variant have been reported in different population as pathogenic, providing support for this variant as a possible candidate to describe the patient’s pheno-type (7, 14, 15).

## Conclusion

To our knowledge, the c.2738dupT mutation in the ASPM gene was first reported and this variant is probably to cause the primary microcephaly reported for this patient. The study might add a new pathogenic variant in mutations of the ASPM gene as causative variant in patients with MCPH and might be helpful in genetic counseling of consanguineous families. However, further studies in other cases should be conducted to clarifying the pathogenic effect of the variant.

## Ethical considerations

Ethical issues (Including plagiarism, informed consent, data fabrication, double publication, etc.) have been completely observed by the authors.
